# Psychometric Evaluation of Large Language Model Embeddings for Personality Trait Prediction

**DOI:** 10.2196/75347

**Published:** 2025-07-08

**Authors:** Julina Maharjan, Ruoming Jin, Jianfeng Zhu, Deric Kenne

**Affiliations:** 1 Department of Computer Science, Kent State University, 800 East Summit Street, Kent, OH, 44242, United States, 1 3305931365; 2 Department of Public Health, Kent State University, Kent, OH, United States

**Keywords:** psychology, Big Five personality, artificial intelligence, large language models, embeddings, deep learning, social media, Reddit

## Abstract

**Background:**

Recent advancements in large language models (LLMs) have generated significant interest in their potential for assessing psychological constructs, particularly personality traits. While prior research has explored LLMs’ capabilities in zero-shot or few-shot personality inference, few studies have systematically evaluated LLM embeddings within a psychometric validity framework or examined their correlations with linguistic and emotional markers. Additionally, the comparative efficacy of LLM embeddings against traditional feature engineering methods remains underexplored, leaving gaps in understanding their scalability and interpretability for computational personality assessment.

**Objective:**

This study evaluates LLM embeddings for personality trait prediction through four key analyses: (1) performance comparison with zero-shot methods on PANDORA Reddit data, (2) psychometric validation and correlation with LIWC (Linguistic Inquiry and Word Count) and emotion features, (3) benchmarking against traditional feature engineering approaches, and (4) assessment of model size effects (OpenAI vs BERT vs RoBERTa). We aim to establish LLM embeddings as a psychometrically valid and efficient alternative for personality assessment.

**Methods:**

We conducted a multistage analysis using 1 million Reddit posts from the PANDORA Big Five personality dataset. First, we generated text embeddings using 3 LLM architectures (RoBERTa, BERT, and OpenAI) and trained a custom bidirectional long short-term memory model for personality prediction. We compared this approach against zero-shot inference using prompt-based methods. Second, we extracted psycholinguistic features (LIWC categories and National Research Council emotions) and performed feature engineering to evaluate potential performance enhancements. Third, we assessed the psychometric validity of LLM embeddings: reliability validity using Cronbach α and convergent validity analysis by examining correlations between embeddings and established linguistic markers. Finally, we performed traditional feature engineering on static psycholinguistic features to assess performance under different settings.

**Results:**

LLM embeddings trained using simple deep learning techniques significantly outperform zero-shot approaches on average by 45% across all personality traits. Although psychometric validation tests indicate moderate reliability, with an average Cronbach α of 0.63, correlation analyses spark a strong association with key linguistic or emotional markers; openness correlates highly with social (*r*=0.53), conscientiousness with linguistic (*r*=0.46), extraversion with social (*r*=0.41), agreeableness with pronoun usage (*r*=0.40), and neuroticism with politics-related text (*r*=0.63). Despite adding advanced feature engineering on linguistic features, the performance did not improve, suggesting that LLM embeddings inherently capture key linguistic features. Furthermore, our analyses demonstrated efficacy on larger model size with a computational cost trade-off.

**Conclusions:**

Our findings demonstrate that LLM embeddings offer a robust alternative to zero-shot methods in personality trait analysis, capturing key linguistic patterns without requiring extensive feature engineering. The correlation between established psycholinguistic markers and the performance trade-off with computational cost provides a hint for future computational linguistic work targeting LLM for personality assessment. Further research should explore fine-tuning strategies to enhance psychometric validity.

## Introduction

### Background

Personality, a foundational construct in psychology, systematically influences human behavior, cognition, and emotion [1]. Its assessment has become indispensable across both academic and applied domains. Contemporary applications range from clinical diagnostics to artificial intelligence (AI)–driven applications such as job candidate evaluation, mental health screening, and personalized recommendation systems [2]. This widespread utility has driven innovation in assessment methodologies, particularly as traditional self-report measures face well-documented limitations regarding scalability and objectivity.

Personality trait assessment has been empirically based mainly on language assessment approaches, grounded in the robust theoretical framework of the Big Five personality model [3]. This empirically validated model organizes personality into 5 core dimensions: openness, conscientiousness, extraversion, agreeableness, and neuroticism. The brief description that characterizes each trait, along with the dimension intended to be measured, is highlighted in Textbox 1.

Textbox 1. **Overview of the Big Five personality traits.**

The Big Five personality traits:

- Openness: represents an individual’s level of curiosity, imagination, and willingness to try new things.
- Conscientiousness: reflects how organized, responsible, and detail-oriented an individual is.
- Extraversion: indicates how outgoing, sociable, and energetic an individual is.
- Agreeableness: measures how cooperative, trusting, and kind an individual is.
- Neuroticism: represents the level of emotional instability, anxiety, and mood swings an individual experiences.

The personality assessment has been long studied by the traditional questionnaire-based approaches by formulating a structured Big Five Inventory (BFI) with the ratings. While survey-based structured questionnaires were the primary source of accessing personality traits, social media platforms, on the other hand, have emerged as particularly valuable sources for personality assessment, offering vast quantities of natural language data that capture authentic behavioral expressions. Unlike controlled laboratory settings or structured surveys, these digital traces provide ecologically valid indicators of personality in real-world contexts. However, the unstructured nature of such data presents significant analytical challenges, including linguistic noise, contextual variability, and potential cultural biases, challenges that demand sophisticated computational solutions.

Nevertheless, the recent advances in AI, particularly in large language models (LLMs), have introduced transformative capabilities for personality assessment in natural language data [4], without the need for traditional BFI questionnaires. These models, as claimed by Lin [5] and Killian and Sun [6], can generate dense, context-aware embeddings that potentially capture deeper psychosocial patterns than traditional psycholinguistic features. Their ability to process natural language at scale while maintaining sensitivity to contextual nuances suggests they may overcome many limitations of previous approaches. In addition to this, the emergence of conversational AI agents such as ChatGPT [7] and DeepSeekAI [8] has revolutionized this domain by enabling zero-shot personality inference, the ability to assess traits without task-specific training. These models can generate preliminary personality profiles through direct prompting, offering immediate applicability in real-world settings.

### Literature Review

#### Overview

The rapid evolution of LLMs has fundamentally transformed personality prediction research, offering new capabilities through both conversational agents [9] and embedding-based approaches [5,6,10-15]. Recent work has established that LLMs not only exhibit emergent personality-like traits but can also emulate specific personality dimensions through targeted prompting techniques [16-18]. This capability stems from their advanced semantic understanding and contextual awareness, as shown by the work of Jiang et al [18], where the examination of personality induction in pretrained models is performed. However, the field lacks consensus on appropriate validation frameworks for these emerging methods. While studies such as by Serapio-García et al [19] and Huang et al [20] have proposed methodologies for quantifying personality in LLMs, where the focus has primarily been on direct model outputs rather than the embedding representations that underlie these predictions.

#### Psychometric Foundations for Computational Assessment

Personality assessment in psychological science relies on rigorous validation of reliability (internal consistency) and validity (convergent or discriminant) [21]. Although recent AI studies have adopted these principles [16-20], most examine only LLMs’ direct responses to structured inventories rather than their embeddings’ psychometric properties. Critical gaps remain in assessing whether embeddings: maintain measurement invariance across demographics [22], align with theoretical constructs beyond surface lexical patterns, and demonstrate consistent reliability in real-world applications. Our work addresses these gaps through comprehensive validation against established psychometric frameworks while correlating embeddings with psychological lexicons (LIWC [Linguistic Inquiry and Word Count] and emotion markers) for theoretical grounding.

#### The Evolution of Text-Based Personality Prediction

The computational assessment of personality has progressed through 3 methodological generations. Early work from 2000 to 2015 established the validity of handcrafted linguistic features, most notably the LIWC dictionary by Pennebaker et al [23], which demonstrated 0.38‐0.42 correlations with the Big Five traits in Facebook (Meta Platforms, Inc) data by Schwartz et al [24]. Subsequent feature sets like the traits detected by Mairesse et al [25] and National Research Council (NRC) Emotion Lexicon [26] enabled more granular analyses, with Park et al [27] achieving 61% accuracy in openness prediction using LIWC+NRC hybrid features. However, these approaches faced well-documented limitations in handling contextual language use [28], particularly for constructs such as neuroticism, where sarcasm and irony distorted predictions [29].

The transformer-based language models revolution since 2018 has focused on BERT variants improving LIWC’s performance on the PANDORA dataset [4]. For instance, Killian and Sun [6] leveraged BERT embeddings on this dataset without linguistic features and presented the state of the art (SOTA) performance score, averaging 526.90 mean square error across all traits. Despite these advances, critical limitations persist in the literature. Serapio-García et al [19] identified 3 major shortcomings: first, the predominant use of generic pretrained models such as vanilla BERT-base, without domain-specific adaptation for personality assessment; second, insufficient controlled comparisons against established psycholinguistic features, as indicated by Tausczik and Pennebaker [28] and Schwartz et al [24]; and third, a concerning absence of measurement invariance testing across demographic groups, despite well-documented lexical biases in language-based assessments [22]. While recent hybrid approaches combining LIWC and BERT features have shown empirical promise [30], these methods often lack robust theoretical foundations explaining their feature integration strategies, potentially limiting their interpretability and generalizability. This gap underscores the need for more principled approaches to combining traditional and neural features in computational personality assessment.

#### Benchmarking Model Architectures

Current research on personality prediction architectures remains disproportionately focused on BERT-family models [31,32], despite emerging evidence of their limitations. While BERT-large achieves strong performance on standard benchmarks, this narrow focus overlooks critical gaps: the efficiency-accuracy trade-offs of larger models such as RoBERTa-base, 125M parameters versus distilled versions, the untested potential of conversational LLMs such as GPT-3.5, whose embeddings may capture richer psychosocial patterns, and the lack of comprehensive evaluations comparing zero-shot versus fine-tuned approaches, assessing cross-model reliability, or examining demographic fairness. These omissions persist despite known biases in language-based personality assessment and the growing need for deployable, equitable systems. Our work addresses these gaps through a systematic comparison of architectures spanning different sizes and training paradigms. Additionally, while zero-shot approaches demonstrate promising scalability, their psychometric properties and depth of analysis remain uncertain compared to more systematic methods. This uncertainty motivates our investigation of whether purpose-trained embedding models can surpass zero-shot capabilities while maintaining practical utility.

Our study addresses these critical gaps through a systematic evaluation of LLM embeddings on a Reddit dataset [4]. Specifically, we aim to investigate the predictive efficacy of LLM embeddings on a psychology domain and analyze their characterization of key psychology dimensions across each trait. In addition to this, we also investigate the benchmark performance-cost trade-offs across model architecture. By grounding this work in the comprehensive Big Five model, we ensure theoretical consistency while advancing computational methods for personality assessment, ultimately contributing to both psychological science and applied AI development. Our specific research questions (RQs) are detailed in Textbox 2.

Textbox 2. **Research questions (RQs).**

RQ1. Do large language models’ (LLMs’) embeddings provide better performance?

RQ2. How do LLM embeddings correlate with psychological constructs to enable personality prediction?

RQ3. Do traditional features (such as Linguistic Inquiry and Word Count [LIWC] and National Research Council emotions), along with feature engineering, improve prediction?

RQ4: How do different LLM embeddings work, and which one is better?

## Methods

### Data Collection and Preprocessing

#### Overview

**Figure 1.**
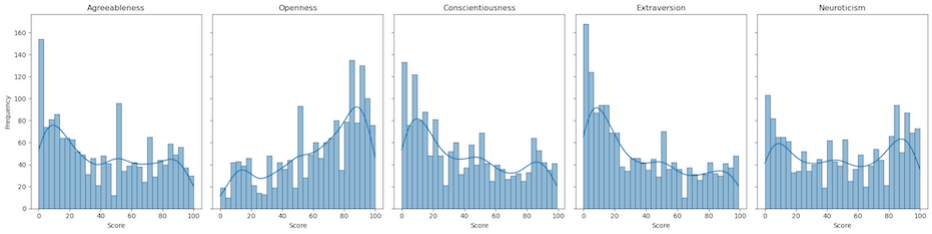
Distribution of the original PANDORA dataset across the Big Five traits.

This study used the PANDORA dataset [4], a large-scale Reddit corpus containing 17 million posts from 10,000 users, annotated with 3 personality assessment models: Myers-Briggs Type Indicator, Big Five, and Enneagram. Given our focus on the Big Five personality traits, we filtered out non-Big Five data, retaining 1568 users and 3 million posts. To ensure linguistic consistency and textual richness, we excluded non-English posts, resulting in 2,830,311 entries, and subsequently removed short posts (fewer than 30 words), leaving 1,008,627 posts. Further refinement involved eliminating duplicate entries, yielding a final dataset of 935,102 posts with an average length of 90 words per post. Importantly, since each post was already associated with prescored Big Five traits at the user level, no additional aggregation of posts per user was required; each post was treated as an independent sample with its corresponding personality labels. The distribution of Big Five trait scores across the dataset is illustrated in Figure 1.

The Big Five personality trait labels in the data were labeled with 3 different scoring methods: scores, percentiles, and descriptions, by the author, ranging from 0 to 100. Exactly, there were 520,065, 268,189, and 145,848 posts with scores, percentiles, and description methods, respectively. The average scores were found to be 43.9 for agreeableness, 63.6 for openness, 39.5 for conscientiousness, 40.4 for extraversion, and 47.9 for neuroticism.

#### Contextual Embedding From LLMs

Embeddings are dense and low-dimensional vector representations of data (words, sentences, or documents) that capture semantic meaning and relationships. When using LLMs such as GPT, BERT, or RoBERTa, embeddings are generated as a byproduct of the model’s internal processing. These embeddings can be used as features for downstream tasks, such as classification, in our case. Unlike traditional vector embedding models such as word2vec, embeddings from LLMs encode the contextual meaning of words in the given sentence corpus. In other words, LLMs can infer a dynamic vector representation of the words, although the words could have multipurpose meanings. These vector representations are generated through their internal architecture based on transformers. The key steps in generating embeddings are tokenization, contextual encoding, and optional pooling.

First, the input is split into tokens and mapped to an initial embedding vector from the model’s embedding layer, known as tokenization. Then, the tokens’ embeddings are processed through the LLM’s transformer architecture, self-attention, and feed-forward networks. Self-attention is a key technique where each token (query) attends to all neighboring tokens (key) to generate an actual result token (value). This step is followed by feedforward neural networks in multiple rounds, where pooling is applied on the final layer. In our experiment, we pooled [CLS] tokens from the final layer of the RoBERTa and BERT models to represent the corresponding contextual sentence embedding.

We generated contextual embeddings using both open-source transformer models and a proprietary API-based conversation AI agent. For open-source models (RoBERTa and BERT), we implemented the 3 key processing steps (tokenization, contextual encoding, and pooling) using the Hugging Face transformers library, specifically using the “roberta-base” and “bert-base-uncased” pretrained models, which produce 768-dimensional embeddings. For comparative analysis, we used OpenAI’s “text-embedding-small-3” model through their paid API, which generates larger 1568-dimensional embeddings. The substantial difference in embedding dimensionality between approaches (768 vs 1568 dimensions) necessitated careful consideration of computational resources, with OpenAI embeddings requiring approximately 48 hours to process our full dataset compared to significantly faster processing times for open-source alternatives. To save the computational cost (both money and inference time) while generating the OpenAI embedding, the generated embedding was backed up to disk to be efficiently used for model development. All other model specifications and corresponding embedding dimensions are detailed in Table 1.

**Table 1.** Embedding dimension across different large language models.

Model architecture	Model name	Dimension	Computation time (hours)	Computation cost (US $)
RoBERTa	roberta-base	768	~2	0
BERT	bert-base-uncase	768	~2	0
OpenAI	text-embedding-small-3	1568	~48	~20

### Persona Classifier (Bidirectional Long Short-Term Memory With Attention)

#### Overview

The classifier module is built on bidirectional long short-term memory (BiLSTM) neural network with an attention mechanism, which is built on 512 hidden units. The 2 independent long short-term memories, left and right, capture the forward and backward information, addressing the limitations of unidirectional models. The forward information ℎ can be calculated as:


$$f_{t}=sigmoid\left(W_{f}[h_{\left(t-1\right)},x_{t}]+b_{f}\right)$$

$$i_{t}=sigmoid\left(W_{i}[h_{\left(t-1\right)},x_{t}]+b_{i}\right)$$

$$C_{t^{\prime }}=tanh\;\left(W_{C}[h_{\left(t-1\right)},x_{t}]+b_{C}\right)$$

$$C_{t}=f_{t}C_{t-1}+i_{t}C_{ti}$$

$$o_{t}=sigmoid\left(W_{o}[h_{t-1},x_{t}]+b_{o}\right)$$

$$h_{f}=o_{t}\;\tanh\;(C_{t})$$

At each time *t*, *x*_*t*_ represents input, *f*_*t*_, *i_t_*, *C_t_*, and *o_t_* represent the forget gate, input gate, cell state, and output gate, respectively. *W* and *b* represent the weight and bias at their corresponding gates. Conversely, the backward ℎ^*b*^ is also calculated. The overall output at *t* is provided by:


$$h_{t}=sigmoid\left(W_{h}[h^{f},h^{b}]+b\right)$$

However, standard BiLSTM often struggles with long-range dependencies, leading us to incorporate an attention mechanism that dynamically assigns importance to relevant textual features. Hence, we incorporated a simple dot product attention layer to capture the weightage information from neighboring vectors; (context, ℎ_*c*_) for each output vector, ℎ_*t*_.


$$Attention\;\left(h_{t},\;h_{c}\right)=\frac{\exp\left(score\;\left(h_{t},\;h_{c}\right)\;\right)}{\sum_{c^{\prime }=1}^{n}\exp\left(score\;\left(h_{t},\;h_{c^{\prime }}\right)\right)}$$

The attention layer computes weights over the hidden states of the BiLSTM, enabling the model to focus on crucial aspects of the input while mitigating information dilution. Finally, the output dense layer generates the score for each sequence vector.

#### Training

We used cuda_12.1.r12.1/compiler.32415258_0 version to train the dataset on a GPU machine. For our model development, we used Sklearn, PyTorch, and Hugging Face libraries. The code base for the entire experimental design can be found on GitHub [33]. We begin with the training by splitting the dataset into 3 splits, 80:10:10 as the training set, validation set, and testing set. Further, for the advanced optimization, we leverage a Grid Search method on the validation dataset to search for the best hyperparameters for our classifiers. The best parameters for each classifier are presented in Table S1 in Multimedia Appendix 1. To prevent overfitting, we applied methods such as dropout with 0.3, early stopping, and batch normalization techniques.

#### Evaluation Metric

Mean squared error (MSE) measures the average squared difference between the predicted values and the actual values. It is a commonly used metric in regression tasks to evaluate the performance of a model. The smaller the MSE, the closer the model’s predictions are. The mathematical notation for MSE is as follows:


$$MSE=\frac{1}{n}\;\;\sum_{i=1}^{n}{\left(y_{i}-\hat{y_{l}}\right)}^{2}$$

Where *n* is the number of data points, and y*_i_* and $\hat{yl}$ represents is the actual and predicted value, respectively, for the *i*th observation.

### Zero-Shot Inference

For the same set of test datasets, we also present zero-shot inference scores. We used the GPTs’ latest model (GPT4o) with a specific prompt design to infer the personality score for each input sequence. We specify the agent as a psychologist expert and give the thorough features regarding Big Five personality traits, followed by the instructions for rating. Each sequence is rated on a scale of 5 (very high) to 1 (very low). The detailed structure of the prompt is presented in Textbox 3.

Textbox 3. **Prompt structure used for zero-shot inference.**

“You are an expert psychologist specializing in personality analysis.

Based on the Big Five Personality Traits model, you will evaluate an individual’s responses to five questions.

Each response reflects different dimensions of personality traits. For each of the Big Five traits, consider the following facets.

Conscientiousness: order, dutifulness, achievement striving, self-discipline, deliberation.

Agreeableness: trust, straightforwardness, altruism, compliance, modesty, tendermindedness.

Neuroticism: anxiety, angry hostility, depression, self-consciousness, impulsiveness, vulnerability.

Openness: fantasy, aesthetics, values.

Extraversion: warmth, gregariousness, assertiveness, excitement-seeking.

Instructions:

Read the individual’s responses to five questions carefully.

For each personality trait, assign a score between 1 (Very Low) and 5 (Very High) based on the themes, tone, and content of the responses.

Each score can be rounded to the nearest tenth, like 3.5.

Scoring Guide:

1: Very Low – The response shows little to no alignment with the trait’s facets. 2: Low – The response shows weak alignment with the trait’s facets.

3: Moderate – The response shows some alignment but not strongly.

4: High – The response strongly aligns with the trait’s facets.

5: Very High – The response shows exceptional alignment with the trait’s facets. {questions_text}

Conversation: {conversation} ### Task:

Analyze the given text for Big Five personality traits and provide the scores in the following format without any explanation or extra words:

“Conscientiousness”: score, “Agreeableness”: score, “Neuroticism”: score, “Openness”: score, “Extraversion”: score”

### Psychometric Test Validation for RQ2

#### Reliability, Convergent, and Divergent Validities on LLMs

In traditional questionnaire-based psychometric tests, researchers often formulate the scales, a set of items to measure a latent construct, as a prior task. For the personality measurement, the psychologist formulates the BFI, consisting of subsequent items to be measured and that undergo a validation check using a psychometric test framework. The validation mainly ought to measure (1) the construct validity to test if scales reflect the underlying construct, (2) reliability (internal consistency) to confirm if the scales are consistent over the same constructs, (3) convergent validity to confirm if the construct correlates with purported indicators (ie, convergent tests) of the same or similar psychological construct, and (4) divergent validity to test the uncorrelatedness with the scores on theoretically unrelated tests.

Similar to item-based measurement, in our study, we aim to measure latent LLMs’ embeddings to validate and check reliability. We performed validations on the predictions resulted from RQ1.

#### Cronbach α for Internal Consistency or Reliability Test

Cronbach α quantifies the internal consistency reliability of a measurement instrument by evaluating the degree to which its items covary relative to their total variance. Mathematically, it is expressed as:


$$\alpha \;=\;\frac{N\;\cdot \;\overset{-}{c}}{\overset{-}{v}+\;\left(N-1\right)\cdot \;\overset{-}{c}}$$

where N is the number of items, $\overset{-}{c}$ is the average interitem covariance, and $\overset{-}{v}$ is the average variance. A higher α (closer to 1) indicates greater consistency among items in measuring the same construct. In our context, we treat LLM-generated embeddings as “items” and compute α to assess whether they reliably capture the target personality trait dimensions. While α≥0.7 is conventionally acceptable for psychological scales, moderate values (0.6‐0.7) may still indicate utility in exploratory research. This adaptation of classical test theory to embedding-based assessment provides a standardized framework for evaluating computational psychometrics.

#### LIWC and Its Correlation With LLM Embedding

LIWC-22, by Pennebaker et al [23], is a powerful linguistic analysis tool that allows researchers to understand the reflection of language on psychological and social dimensions. The tool helps in analyzing the texts by mapping the words in the text to prevalidated dictionaries of words from psychological categories such as emotion, cognition, social process, and social references. In the personality trait assessment, the tool has been a primary choice among many researchers given its powerful psychological dictionary. In our work, we used this software to generate the static linguistic features (a total of 119 features) on the studied data.

To assess the correlation of LLM embedding with LIWC, we begin by generating LIWC features on the test dataset from RQ1. Due to the sparsity and redundant nature of features in LIWC, we run Lasso Regression to retrieve the highly important features for each personality trait. Similarly, for the high-dimensional embeddings, we performed principal component analysis (PCA) to reduce dimensions where principal components were chosen from 5 to 20 to match with the retrieved features from the LIWC.

#### NRC Emotion Lexicon and Its Correlation With LLM Embedding

NRC Emotion Lexicon [26] is a widely validated and extensively used lexical resource in sentiment and emotion analysis. It is a crowdsourced word-emotion association lexicon that categorizes English words into 8 basic emotions (joy, sadness, anger, fear, trust, disgust, surprise, and anticipation) and 2 sentiment polarities (positive and negative). Each word in the lexicon is annotated based on its association with these emotions and sentiments, providing a granular approach to emotion detection in text.

In our analysis, we applied the NRC Emotion Lexicon to study the correlation of emotions in condensed LLMs embedding to assess the emotional feature encapsulation in LLMs. Similar to LIWC, we begin by generating these 10 NRC emotion features (anger, anticipation, disgust, fear, joy, negative, positive, sadness, surprise, and trust) on the test dataset from RQ1. Additionally, we iteratively performed PCA on the embedding to reduce the dimension from 3 to 10 to perform correlation with the extracted emotion features.

### Psycholinguistic Features and Feature Engineering for RQ3

#### Overview

In addition to evaluating the classifier on only LLM embedding, we additionally assessed the model by training on combined psycholinguistic features with advanced feature engineering. In total, we experimented in 4 different settings: only linguistic features, only contextual embedding, all linguistic features with contextual embedding, and optimal linguistic features with contextual embedding, where the contextual embedding model was OpenAI.

#### Combined Psycholinguistic Features

We combined all linguistic features; LIWC features (119 features), NRC emotion features (10 features), NRC valence, arousal, and dominance (3 features) and VADER (Valence Aware Dictionary and Sentiment Reasoner) sentiment (1 feature), a total of 133 features. These features were further processed with advanced feature engineering and feature selection methods to assess the model performance.

#### Feature Engineering

##### Feature Scaling or Normalization, and Imputation

Feature Scaling is the process of transforming the features to a common scale. Scaling features often help the optimization algorithms to converse faster, as the gradient updates will be dominated by larger magnitudes otherwise. Further, scaling also ensures that all the features contribute equally to the model’s learning process, preventing bias toward features with larger values. In our work, we used standard scaling with a mean (μ) of 0 and an SD (σ) of 1, as $X_{scaled}=\frac{X-\mu }{\sigma }.$ Imputation is the process of handling missing values in the dataset, which leads to bias or incorrect model training otherwise. We have replaced the missing values with the mean value of the feature column.

##### Feature Selection

Feature selection is the process of identifying and selecting the most relevant features for building a model such as to speed up training and reduce overfitting. In our work, we experiment with a combination of irrelevant filter, redundant filter, and a standard filter selection method, mutual information.

Our combination technique began with an irrelevant filter method to remove filters that have little or no predictive power per the target. For this, we used a variance threshold method to filter features with very low variance. Then, a redundant filter method was used to remove highly correlated features by using the correlation matrix method with different sets of thresholds: 0.25, 0.5, and 0.6, as shown in Table S2 in Multimedia Appendix 1. Finally, we applied the mutual information method to measure the information between features and target variables and selected the features based on the ranking of the mutual information scores.

### Training

Our comparative analysis under different settings is based on binary classification, which required transformation of continuous personality scores into discrete classes. For this, we normalized all trait scores using MinMaxScaler to ensure consistent scaling across dimensions. The median was selected as the binarization threshold based on its established use in psychological research for creating balanced high or low trait groups when clinical cutoffs are unavailable. This approach mitigates class imbalance issues while maintaining interpretability. The resulting class distributions across all 3 scoring criteria are presented in Table 2.

**Table 2.** Binary class distribution.

Label	Agreeableness	Openness	Conscientiousness	Extraversion	Neuroticism
0	448,041	445,127	424,105	407,798	456,137
1	487,061	489,975	510,997	527,304	478,965

We used the same model architecture as in RQ1 with an additional sigmoid function at the final layer. The sigmoid activation module on the final layer ensured the score was between 0 and 1. Simultaneously, we used binary cross-entropy as a loss function to train the model. For the true output y*_i_* and total input samples *N*, the loss function is calculated as:


$$BCE\;(y,\hat{y})=-\frac{1}{N}\sum_{i=1}^{N}\left[y_{i}\cdot \log\;({\hat{y}}_{i})+(1-y_{i})\cdot \log\;(1-{\hat{y}}_{i})\right]$$

Similar to the regression task, the hyperparameters were chosen using grid search for all 5 classifiers, and methods such as batch normalization, dropout layer, and early stopping were applied to prevent overfitting. Likewise, the training was validated using k-fold validation techniques with 5 folds to ensure a reliable estimate of the model’s generalization ability.

### Evaluation Metric

We used the area under the receiver operating characteristic curve (AUROC) to measure the performance of binary classification models. It quantifies the model’s ability to discriminate between positive and negative classes across all possible classification thresholds. The ROC curve plots the true positive rate (TPR) against the false positive rate (FPR) at varying thresholds, and the AUROC represents the area under this curve.

TPR measures the proportion of actual positive data points that a model correctly identifies as positive. It is calculated as:


$$TPR=\frac{TP}{\left(TP+TN\right)}$$

FPR measures the proportion of negative data points that a model incorrectly identified as positive. It is calculated as:


$$FPR=\frac{FP}{TN+FP}$$

Here, TP and TN refer to the count of data points correctly identified as positive and negative, respectively, while FP refers to the count of data points incorrectly identified as positive.

With the above TPR and FPR values, AUROC is computed as:


$$AUROC=\int_{0}^{1}TPR\;(FPR)\;d(FPR)$$

Generally, an AUROC value equal to 1 is considered a perfect classifier, and a value above 0.75 is considered a good classifier.

### Ethical Considerations

This study was conducted using the publicly available, anonymized PANDORA dataset of Reddit posts, which contains no identifiable user information. Ethical approval was obtained from the Institutional Review Board of Kent State University (IRB protocol #19‐353). The research complied with Reddit’s API terms of service and adhered to the ethical guidelines established by the original dataset creators. As the analysis involved only deidentified text data without human participant interaction, it posed no additional privacy risks. Funding support was provided in part by the Substance Abuse and Mental Health Administration (SAMHSA grant #H79SP081502). The funder had no role in study design, data collection, analysis, or publication decisions. All methods focused exclusively on computational analysis of linguistic patterns for personality traits, with no attempt to identify individuals or infer sensitive personal information.

## Results

### RQ1. Do LLMs’ Embeddings Provide Better Performance?

Our primary work begins with experimenting with the variant of LLMs’ embedding (RoBERTa and OpenAI) to perform Big Five personality traits prediction on our custom model (BiLSTM with attention). To compare with SOTA (Killian and Sun) [6], we have approached the regression task with MSE as an evaluation metric. In addition to training the model using LLM embedding as features, we also experiment with zero-shot inference on the same test data. The performance of the experimented models with SOTA is presented in Table 3.

**Table 3.** Performance measured by MSE^a^ across different LLM^b^ embeddings and zero-shot.

	OPN^c^	CON^d^	EXT^e^	AGR^f^	NEU^g^	Average
BERT (Killian and Sun [6])	424.9	550.32	513.22	585.18	581.91	531.11
RoBERTa	496.12	641.38	610.37	702.71	641.64	618.44
OpenAI^h^	417.87	546.43	510.37	599.50	560.33	526.90
Zero-shot (GPT4.o)	668.81	767.038	848.35	919.47	624.57	765.647

a MSE: mean squared error.

b LLM: large language model.

c OPN: openness.

d CON: conscientiousness.

e EXT: extraversion.

f AGR: agreeableness

g NEU: neuroticism.

h The mean squared error value indicates the best performance across all personality traits.

Our experiments demonstrate that OpenAI embeddings trained on a BiLSTM model achieve superior performance (average MSE=526.90) compared to both other embedding approaches (including SOTA; average MSE=531.11) and zero-shot inference (average MSE=765.64). This substantial performance gap (45% lower error) not only confirms the advantage of custom-trained embeddings over direct zero-shot application but also suggests potential misinterpretation risks when relying solely on untuned LLM outputs for personality assessment.

### RQ2. How Do LLM Embeddings Correlate With Psychological Constructs to Enable Personality Prediction?

#### Overview

We extended our experiment from RQ1 to establish a validation of our result. Thus, we formulated a test framework inspired by the traditional psychometric framework to assess reliability or convergent and divergent validation on LLM embedding. Essentially, we performed validations on the predictions resulting from RQ1.

#### Reliability, Convergent, and Divergent Validities on LLMs

##### Cronbach α for Internal Consistency or Reliability Test

Cronbach α is a way of assessing reliability by comparing the amount of shared variance, or covariance, among the items making up an instrument to the amount of overall variance. Here, we evaluate the Cronbach α on the LLMs embeddings across all 5 personality traits and observed the score above 0.6 in all personality traits except conscientiousness (Table 4).

**Table 4.** Cronbach score for large language models embedding across the Big Five traits.

Big traits	Cronbach score
Openness	(0.6637411384440958, array([0.648, 0.679]))
Conscientiousness	(0.5742894806444646, array([0.547, 0.601]))
Extraversion	(0.6187154356717698, array([0.597, 0.64]))
Agreeableness	(0.6476180569410122, array([0.625, 0.669]))
Neuroticism	(0.6376958477093122, array([0.594, 0.679]))

##### Correlation Score for Convergent and Divergent Validity Test

**Figure 2.**
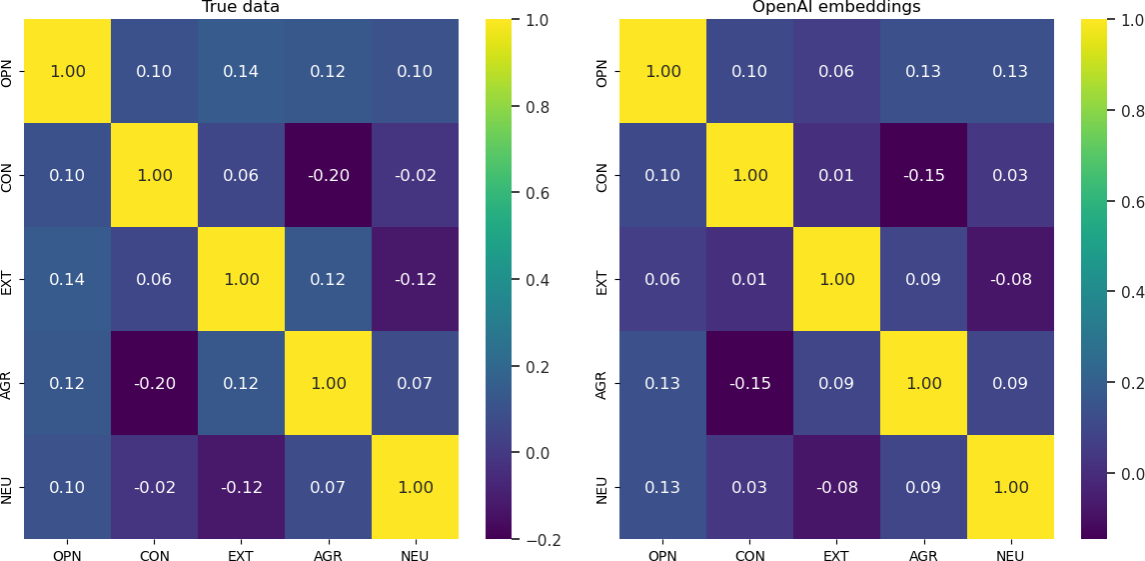
Heatmap between Big Five traits (**A**) personality score in the true data and (**B**) OpenAI embeddings. AGR: agreeableness; CON: conscientiousness; EXT: extraversion; NEU: neuroticism; OPN: openness.

Additionally, we calculated the correlation scores among Big 5 personality traits in 2 different ways: the actual score correlation and the embedding correlations across personality traits, as shown in Figure 2, on the left and right, respectively. Ideally, openness and agreeableness, and conscientiousness and extraversion are considered correlated, while neuroticism is often unrelated to all other traits per a study by Oz [34]. Our correlation results in both settings demonstrate a similar result. Additionally, the correlation score in both results has the same direction except for conscientiousness and neuroticism, where −0.02 was observed in true data while with 0.003 in the LLMs embeddings score.

Our psychometric validation of LLM embeddings revealed strong alignment with traditional personality measurement principles. The embeddings demonstrated acceptable internal consistency (Cronbach α>0.6) for 4 of the 5 traits, with only conscientiousness falling slightly below this threshold. Correlation analyses showed remarkable convergence between embedding-derived traits and theoretical expectations: openness-agreeableness and conscientiousness-extraversion showed positive relationships, while neuroticism remained largely independent of other traits, mirroring patterns in ground-truth data (Figure 2). The sole deviation appeared in the conscientiousness-neuroticism relationship, where embeddings showed near-0 correlation (0.003) versus the slight negative association (−0.02) in original scores, suggesting minimal but measurable divergence in how these traits relate in the embedding space.

### Correlation Between LIWC Features and Reduced LLM (OpenAI) Embedding

**Figure 3.**
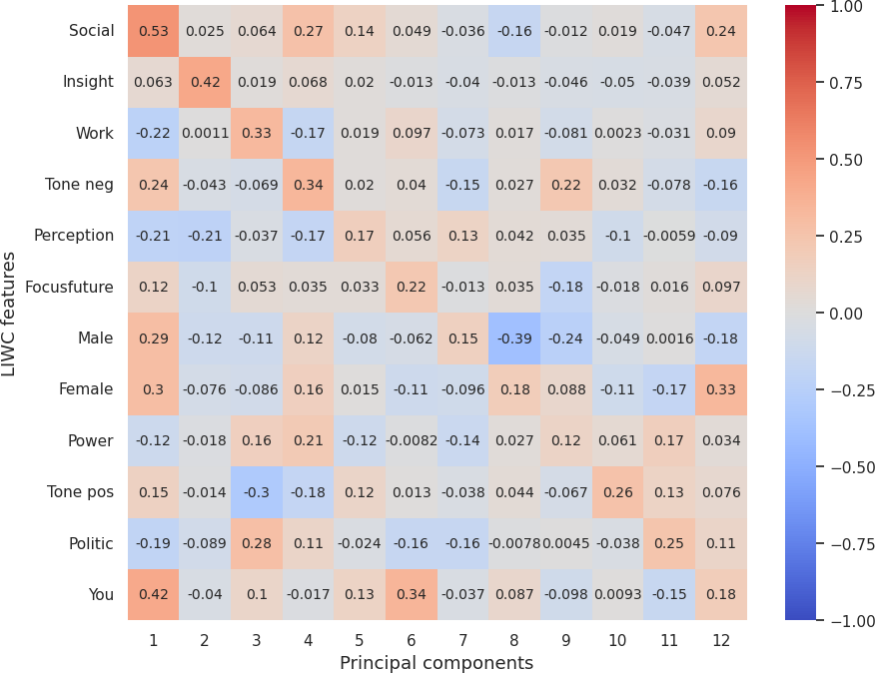
Correlation score between top 12 LIWC features and OpenAI embedding (reduced PCA components) for openness. LIWC: Linguistic Inquiry and Word Count; PCA: principal component analysis; pos: positive.

**Figure 4.**
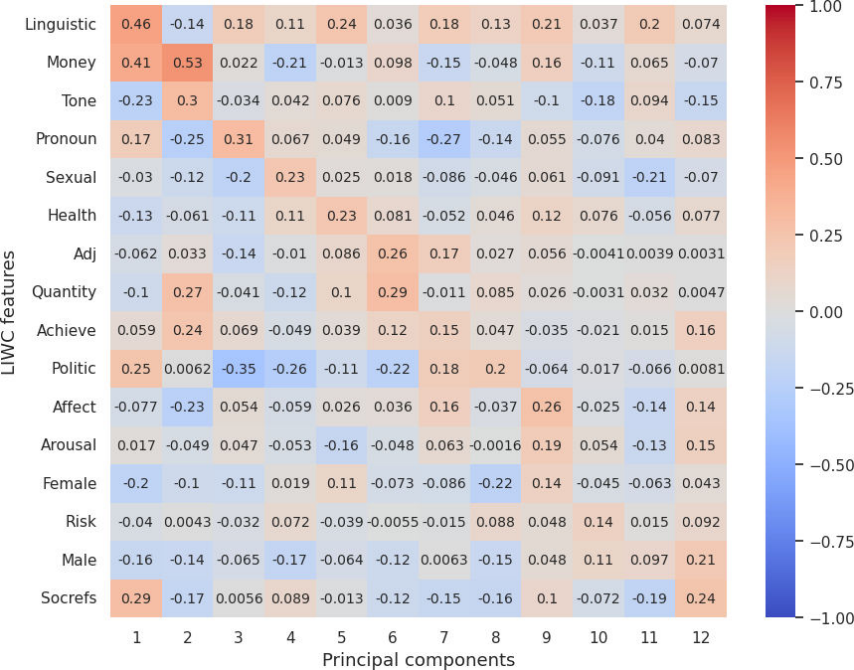
Correlation score between top 12 LIWC features and OpenAI embedding (reduced PCA components) for conscientiousness. Adj: Adjective; LIWC: Linguistic Inquiry and Word Count; PCA: principal component analysis.

**Figure 5.**
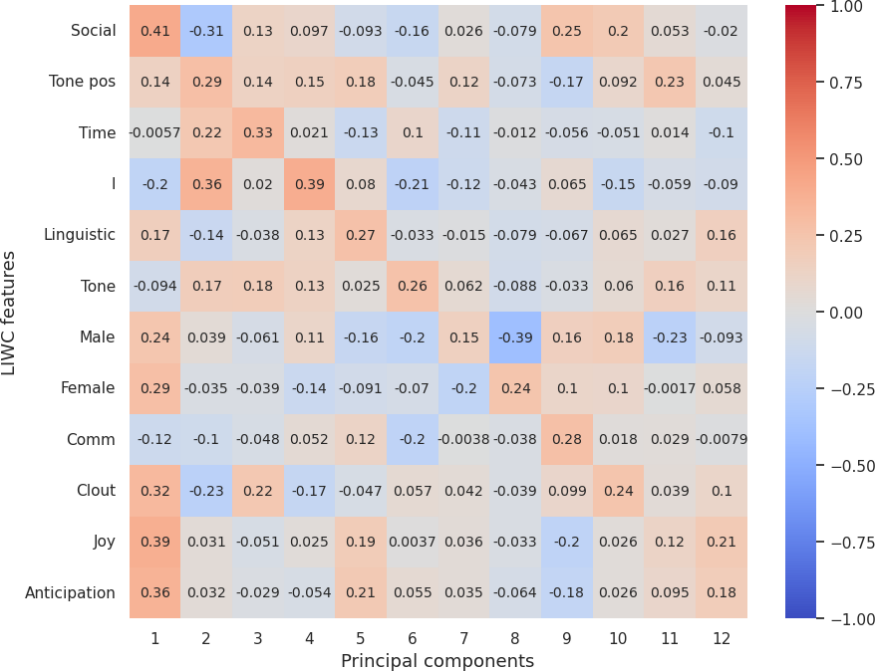
Correlation score between top 12 LIWC features and OpenAI embedding (reduced PCA components) for extraversion. LIWC: Linguistic Inquiry and Word Count; PCA: principal component analysis.

**Figure 6.**
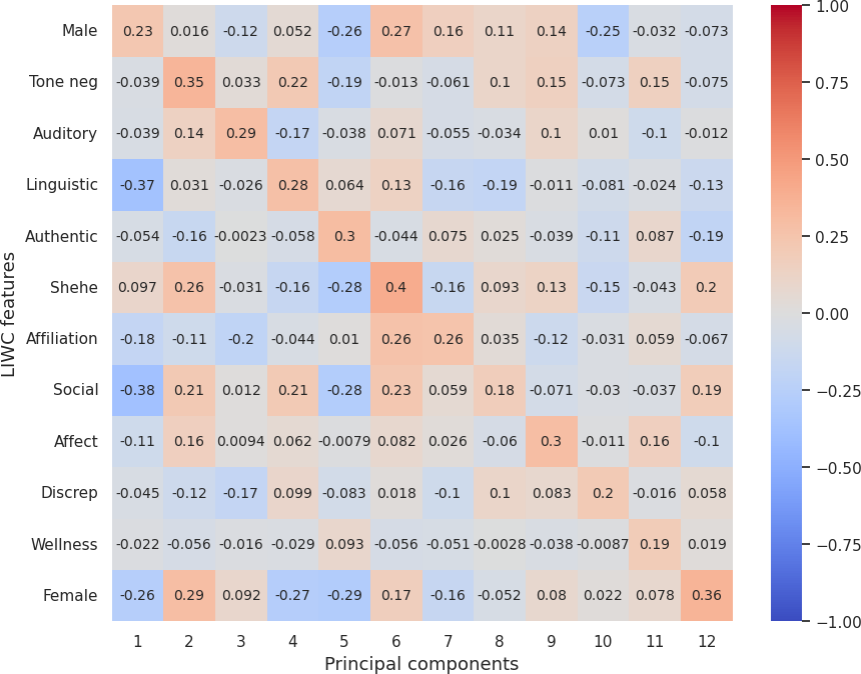
Correlation score between top 12 LIWC features and OpenAI embedding (reduced PCA components) for agreeableness. Discrep: discrepancy; LIWC: Linguistic Inquiry and Word Count; neg: negative; PCA: principal component analysis.

**Figure 7.**
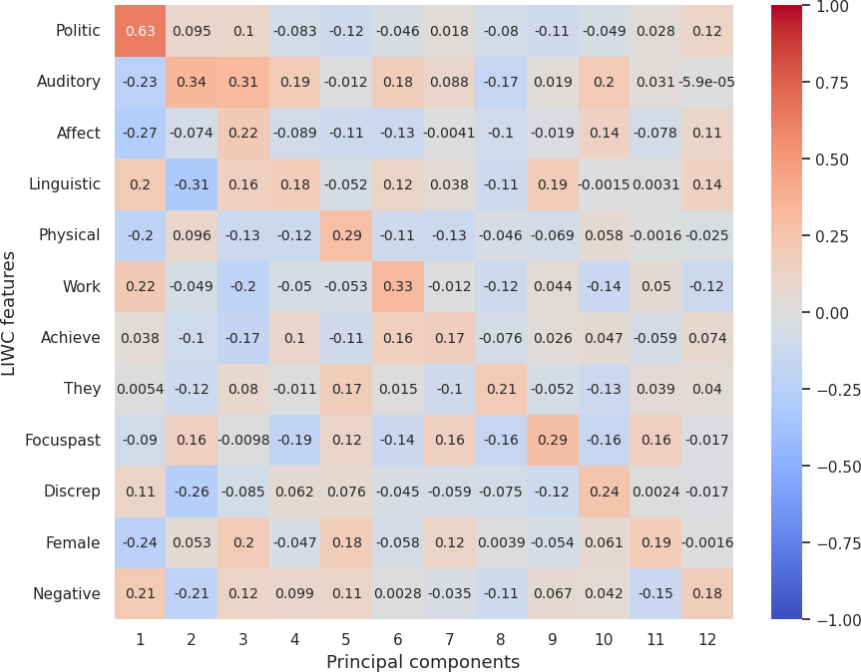
Correlation score between top 12 LIWC features and OpenAI embedding (reduced PCA components) for neuroticism. Discrep: discrepancy; LIWC: Linguistic Inquiry and Word Count; PCA: principal component analysis.

Our analysis from correlation Figures 3-7 highlights the top 12 LIWC features’ association with the top 12 principal components across each personality trait. For the openness and extraversion traits, social feature was identified as a key feature with the correlation score of 0.53 and 0.41, respectively. Likewise, linguistic features have been highlighted as a key feature for conscientiousness with a score of 0.46, and politic features for neuroticism with a score of 0.63. However, for agreeableness, the correlation score does not account for high correlation to any specific feature, although the result shows a high correlation with the use of pronouns (she and he, denoted by “shehe”) with a 0.4 score.

### Correlation Between Emotion Features and Reduced LLM (OpenAI) Embedding

**Figure 8.**
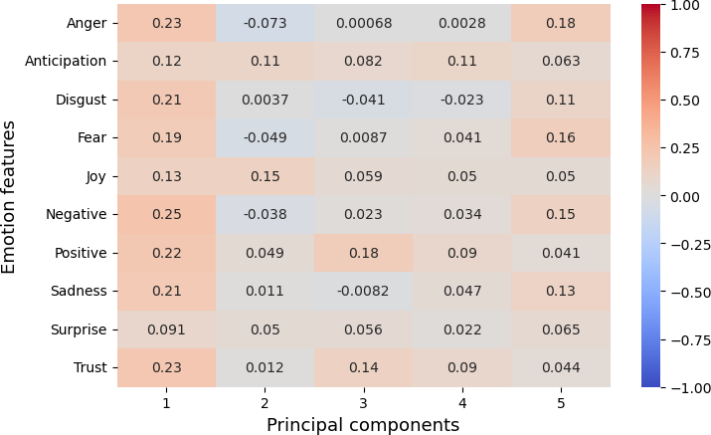
Correlation between emotion features and OpenAI embedding (reduced PCA components). PCA: principal component analysis.

The result from Figure 8 shows that emotion features are captured in only a single dimension (first principal axis) rather than multiple dimensions. However, the third dimension highlights the semantic relation between positive emotions, where trust, positivity, and anticipation were observed with a moderate correlation. Likewise, the negative emotions were observed in the fifth dimension with a moderate correlation between anger, disgust, fear, negativity, and sadness.

Overall, the correlation with LIWC and emotions features demonstrated that the embeddings essentially capture the key linguistic properties required for personality assessment and provide evidence on the reliability of LLM embeddings for personality prediction.

### RQ3. Do Traditional Features (Such as LIWC and NRC Emotions), Along With Feature Engineering, Improve Prediction?

In this section, we analyze the impact of psycholinguistic features along with LLMs embedding on the personality prediction task under 4 different settings: only linguistic features, only contextual embedding, both linguistic features and contextual embeddings, and optimal linguistic features (obtained from feature engineering) and contextual embeddings. For all settings, a BiLSTM with attention model was trained for a binary classification task. The performance scores of the experiment across all settings are presented in Table 5.

**Table 5.** Binary classification performance AUROC^a^ under 4 different settings

	Only linguistic features	Only contextual (OpenAI) embedding	All linguistic features with contextual embedding	Optimal linguistic features with contextual (OpenAI) embedding
Openness	0.65	0.82	0.64	0.82
Conscientiousness	0.63	0.82	0.62	0.81
Extraversion	0.65	0.83	0.65	0.80
Agreeableness	0.66	0.83	0.66	0.83
Neuroticism	0.65	0.82	0.64	0.81

a AUROC: area under the receiver operating characteristic curve.

The result shows the best performance under 2 settings: only contextual embedding and optimal linguistic features with contextual embedding. While feature engineering showed nearly equal performance because without linguistic features, using all linguistic features, in fact, had a negative impact on the model performance. This highlights that the use of contextual embeddings has a greater impact than all.

### RQ4. How Different LLM Embeddings Work, and Which One Is Better?

In the final phase of our analysis, we evaluate the systematic comparison of 3 LLM embedding approaches: RoBERTa (roberta-base), BERT (bert-base-uncased), and OpenAI (text-embedding-small-3). These models vary in architecture, scale, and computational requirements. BERT contains 110M parameters, RoBERTa is slightly larger at 125M, and OpenAI’s model is significantly larger at 355M parameters. Embedding dimensions also differ, with RoBERTa and BERT producing 768-dimensional vectors, while OpenAI generates higher-dimensional embeddings at 1536 dimensions. Computational costs further distinguish these models: OpenAI’s embeddings incurred an approximate cost of US$ 20 for our dataset and took 48 hours to generate embeddings for all datasets and required additional storage space to reduce runtime cost, whereas RoBERTa and BERT required no monetary cost and storage space, but demanded nearly 2 hours for inferencing runtime.

Performance evaluation using the AUROC for binary classification yielded insightful comparisons, as shown in Table 6. OpenAI’s embeddings demonstrated consistent superiority, achieving a mean AUROC score of 0.824 (SD 0.0055) across all personality traits. While this performance advantage is clear, RoBERTa’s competitive results (ranging from 0.80 to 0.82 AUROC) suggest meaningful implications for practitioners considering the cost-performance trade-off. The observed pattern indicates that while model scale and embedding dimensionality contribute to predictive accuracy, the decision between proprietary and open-source solutions must weigh computational resources against marginal performance gains.

**Table 6.** Binary classification performance AUROC^a^ in different large language models’ embeddings

	RoBERTa	BERT	OpenAI^b^
Openness	0.80	0.74	0.82
Conscientiousness	0.80	0.73	0.82
Extraversion	0.81	0.75	0.83
Agreeableness	0.82	0.73	0.83
Neuroticism	0.80	0.74	0.82

a AUROC: area under the receiver operating characteristic curve.

b The hightest area under the receiver operating characteristic curve is observed with OpenAI embedding across all personality traits.

## Discussion

### Primary Results

In this study, we explored the potential of LLMs’ embeddings in assessing personality traits using the well-labeled PANDORA Reddit dataset. Our findings demonstrate that LLMs’ embeddings effectively capture nuanced psycholinguistic properties essential for personality trait identification, offering a robust alternative to traditional methods. From RQ1, we observed that LLMs’ embeddings, when paired with a simple deep learning model (BiLSTM with attention), significantly outperformed zero-shot inference results. Specifically, conversational AI embeddings, OpenAI, showed superior performance compared to frozen embeddings such as BERT and RoBERTa, though the margin of improvement was not substantial. This suggests that while LLMs’ embeddings are highly effective, the choice of model architecture plays a nuanced role in performance.

For RQ2, our psychometric validity analysis revealed an average Cronbach α of 0.63, indicating moderate reliability across personality traits. Additionally, the correlation between embeddings and the original dataset’s personality traits highlighted the ability of LLMs’ embeddings to preserve the underlying structure of the data. Further, dimensionality reduction via PCA and correlation analysis with LIWC and emotion features demonstrated that embeddings effectively encapsulate relevant psycholinguistic information, by showcasing key features such as social for extraversion and openness, politic for neuroticism, linguistic for conscientiousness, and pronouns for agreeableness. Additionally, the polarity of emotion in a certain dimension clearly evidences that embeddings pose the claim.

Regarding RQ3, our experiments with feature engineering revealed that LLMs’ embeddings consistently outperformed linguistic features alone. Notably, combining linguistic features with embeddings did not yield significant improvements, suggesting that LLMs’ embeddings inherently capture the necessary linguistic and contextual information for personality prediction. This finding highlights the potential of LLMs to reduce reliance on domain-specific feature engineering, making them a powerful tool for psychological research.

Finally, in RQ4, we compared the performance of different LLMs’ embeddings (RoBERTa, BERT, and OpenAI) and found that OpenAI and RoBERTa embeddings achieved similar scores, with an average AUROC of 0.81 across all personality traits. While OpenAI embeddings slightly outperformed RoBERTa in some cases, the difference was not marginal. Given the computational and financial costs associated with generating OpenAI embeddings, our results suggest that RoBERTa, as an open-source model, provides a cost-effective and equivalent alternative for personality prediction tasks. This finding highlights the practical utility of open-source models such as RoBERTa, especially in resource-constrained settings, without compromising on performance.

### Implications for Computational Social Science and Psychology

Our results carry significant implications for computational social science and psychology. First, we demonstrate that LLM embeddings enable scalable, naturalistic personality assessment, circumventing the limitations of traditional survey methods. This scalability opens avenues for large-scale sample studies across diverse populations, enhancing generalizability in psychological research. Second, the alignment between embedding-derived features and established psycholinguistic markers such as LIWC and emotions supports their theoretical validity, suggesting that LLMs can complement or even augment existing psychological frameworks.

Practically, the trade-offs between proprietary and open-source models highlight the importance of cost-benefit considerations in research design. While OpenAI embeddings showed marginal gains, their computational and financial overhead may not justify their use in large-scale applications, where RoBERTa offers a performant, accessible alternative. This insight is particularly relevant for resource-limited settings, such as academic or public health research, where budget constraints often dictate methodological choices.

Overall, our results underscore the efficacy of LLMs’ embeddings in personality prediction and their ability to generalize across diverse psycholinguistic contexts. The consistent performance of embeddings highlights their potential as a leading tool for psychological and personality-related research, while also emphasizing the importance of model selection in achieving optimal results.

### Limitations

Our study has several important limitations that merit careful consideration. As the moderate reliability scores (Cronbach α=0.63) indicate that while LLM embeddings show promise for personality assessment, more rigorous psychometric validation through methods such as confirmatory factor analysis or item response theory is needed before clinical or organizational application. This is particularly crucial given the risks of misinterpretation when these tools are used by nonexperts.

The research was constrained by several methodological factors that could impact validity. First, we exclusively examined OpenAI’s embeddings without comparison to other advanced conversational AI systems (such as Claude or DeepSeekAI) or human-rated benchmarks, potentially masking model-specific biases. Second, the lack of clinical validation data is particularly noteworthy, as personality assessment tools often require careful calibration for diagnostic use; our findings from Reddit data may not generalize to clinical populations or other platforms. Third, our reliance on the PANDORA dataset alone raises questions about whether these models might perpetuate or amplify existing biases when applied to different demographic groups or other data sources such as Facebook’s MyPersonality or Twitter’s (subsequently rebranded X) Myers-Briggs Type Indicator personality dataset.

Practical and ethical challenges emerged from our findings. The substantial computational and financial costs of proprietary models create accessibility barriers while potentially exacerbating inequities in research capabilities. More fundamentally, the “black box” nature of LLM embeddings creates interpretability challenges that could lead to misuse in high-stakes scenarios such as hiring or mental health screening without proper safeguards.

### Future Works

The identified limitations point to several important avenues for advancing LLM-based personality assessment. More robust psychometric validation using confirmatory factor analysis and item response theory could strengthen the clinical applicability of these methods, particularly for diagnostic purposes. Comparative studies incorporating newer proprietary models such as Claude, DeepSeekAI, and open-source language models like LLAMA-2, along with human-rated benchmarks, would provide crucial insights into their relative performance across diverse populations and datasets. The integration of multimodal data sources, including visual content and behavioral metadata, presents opportunities to enhance assessment accuracy while improving model interpretability. Developing standardized evaluation protocols that account for cultural variability and computational efficiency remains essential for ensuring both the scientific validity and practical utility of these approaches. Addressing these challenges through interdisciplinary collaboration could significantly advance the field while mitigating risks of bias and misuse in real-world applications.

### Conclusions

This study establishes the efficacy of LLM embeddings in personality trait prediction, with both OpenAI and RoBERTa embeddings demonstrating superior performance compared to zero-shot inference and conventional linguistic feature-based approaches. Our rigorous validation framework, incorporating psychometric analysis and comparative model evaluation, provides robust evidence for the utility of these embeddings in computational personality assessment. While OpenAI embeddings achieved marginally better performance, RoBERTa emerged as a computationally efficient alternative with comparable predictive capability.

For AI researchers, these findings highlight critical trade-offs between model performance and computational resources that must be considered in practical implementations. Psychologists may find value in these tools as complementary approaches to traditional assessment methods, pending further clinical validation. Linguists can leverage these findings to investigate deeper relationships between language understanding and personality expression in LLMs. Future research directions should prioritize expanding validation across diverse datasets, incorporating emerging model architectures, and developing robust bias mitigation strategies.

## Supplementary material

10.2196/75347 Multimedia Appendix 1 Additional experimental settings and results to support this paper’s investigation.

## Abbreviations

AI: artificial intelligence
AUROC: area under the receiver operating characteristic curve
BFI: Big Five Inventory
BiLSTM: bidirectional long short-term memory
FPR: false positive rate
LIWC: Linguistic Inquiry and Word Count
LLM: large language model
MSE: mean squared error
NRC: National Research Council
PCA: principal component analysis
RQ: research question
SOTA: state of the art
TPR: true positive rate
VADER: Valence Aware Dictionary and Sentiment Reasoner
