# Supplementary material for: Psychometric Evaluation of Large Language Model Embeddings for Personality Trait Prediction
Source: J Med Internet Res. 2025 Jul 8;27:e75347. doi: 10.2196/75347 (PMC12262148; doi:10.2196/75347)
Supplement: Multimedia Appendix 1 [file jmir-v27-e75347-s001.docx]

**Do Large Language Models Really Understand Personality? A Test of Embeddings Versus Zero-Shot**

**Table S1.** Hyperparameter search for BiLSTM.

|  | Parameter  Space | OPN | CON | EXT | AGR | NEU |
| --- | --- | --- | --- | --- | --- | --- |
| Hidden  Size | [128, 256] | 128 | 256 | 128 | 256 | 128 |
| Learning rate | [0.01, 0.001,  0.0001,  0.00001] | 0.001 | 0.001 | 0.001 | 0.001 | 0.0001 |
| Batch size | [8,16] | 8 | 8 | 16 | 16 | 16 |
| Dropout rate | [0.2, 0.3,  10.5] | 0.5 | 0.3 | 0.3 | 0.3 | 0.3 |
| Epochs | 32 with early stopping | 32 with early stopping | 32 with early stopping | 32 with early stopping | 32 with early stopping | 32 with early stopping |

**Table S2.** Feature selection under different thresholds experimented for RQ3.

| 0.25 | 0.4 | 0.5 | 0.6 |
| --- | --- | --- | --- |
| Correlated dropped: 101 (908432, 32) Index(['Segm  ent', 'WC',  'Analytic',  'Clout', 'Tone',  'WPS',  'BigWords',  'we',  'they',  'adj', 'allnone',  'memory',  'Affect',  'Lifestyle',  'home',  'Physical',  'substances',  'need',  'acquire',  'lack', 'fulfill',  'fatigue',  'risk',  'curiosity',  'auditory',  'feeling',  'focuspast',    'Conversation  ', 'filler',  'QMark',  'Exclam',  'Emoji'] | Correlated dropped: 68  (908432, 65)  Index(['Segment'  , 'WC', 'Analytic',  'Clout', 'Tone',  'WPS',  'BigWords', 'Dic',  'we', 'shehe',  'they', 'det',  'number', 'conj',  'negate', 'adj',  'quantity',  'allnone', 'cause',  'discrep',  'certitude',  'memory',  'Affect',  'swear',  'prosocial',  'polite', 'moral',  'family', 'friend',  'Culture',  'Lifestyle',  'home', 'relig',  'Physical',  'wellness',  'substances',  'sexual', 'death',  'need', 'acquire',  'lack', 'fulfill',  'fatigue',  'reward', 'risk',  'curiosity',  'allure',  'Perception', | Correlated dropped: 52 (908432, 81)  Index(['Segment  ', 'WC',  'Analytic',  'Clout',  'Authentic',  'Tone', 'WPS',  'BigWords',  'Dic', 'we',  'shehe', 'they',  'det', 'number',  'prep',  'adverb',  'conj', 'negate',  'adj', 'quantity',  'Drives',  'achieve',  'allnone',  'cause', 'discrep',  'certitude',  'memory',  'Affect',  'emo_anx',  'emo_sad',  'swear',  'prosocial',  'polite', 'conflict',  'moral',  'family', 'friend',  'Culture',  'ethnicity',  'Lifestyle',  'leisure',  'home', 'relig',  'Physical', | Correlated dropped: 34 (908432, 99)  Index(['Segment  ', 'WC',  'Analytic',  'Clout',  'Authentic',  'Tone', 'WPS',  'BigWords',  'Dic', 'i', 'we',  'you', 'shehe',  'they', 'ipron',  'det',  'article',  'number', 'prep',  'auxverb',  'adverb', 'conj',  'negate',  'adj',  'quantity',  'Drives',  'achieve',  'Cognition',  'allnone',  'insight',  'cause',  'discrep',  'tentat',  'certitude',  'differ',  'memory',  'Affect',  'emo_anx',  'emo_anger',  'emo_sad',  'swear', |

|  | 'attention',  'visual',  'auditory',  'feeling', 'time',  'focuspast',  'focusfuture',  'Conversation',  'nonflu', 'filler',  'AllPunc',  'Period',  'Comma',  'QMark',  'Exclam', 'Emoji',  'Arousal'] | 'wellness',  'substances',  'sexual',  'death', 'need',  'want', 'acquire',  'lack', 'fulfill',  'fatigue',  'reward', 'risk',  'curiosity',  'allure',  'Perception',  'attention',  'motion', 'visual',  'auditory',  'feeling', 'time',  'focuspast',  'focuspresent',  'focusfuture',  'Conversation',  'nonflu',  'filler',  'AllPunc',  'Period',  'Comma',  'QMark',  'Exclam',  'Apostro',  'Emoji',  'Valence',  'Arousal',  'Dominance'] | 'prosocial',  'polite',  'conflict',  'moral', 'family',  'friend',  'female',  'Culture',  'ethnicity',  'Lifestyle',  'leisure', 'home',  'money',  'relig', 'Physical',  'health', 'illness',  'wellness',  'mental',  'substances',  'sexual', 'food',  'death', 'need',  'want', 'acquire',  'lack',  'fulfill', 'fatigue',  'reward', 'risk',  'curiosity',  'allure',  'Perception',  'attention',  'motion',  'visual',  'auditory',  'feeling',  'time',  'focuspast',  'focuspresent',  'focusfuture',  'Conversation',  'assent',  'nonflu', 'filler',  'AllPunc',  'Period',  'Comma',  'QMark',  'Exclam',  'Apostro',  'Emoji',  'Valence', |
| --- | --- | --- | --- |
|  |  |  | 'Arousal',  'Dominance',  'sent_score'] |

## Experiment results in six settings

*Settings:*

**Setting S1.** Only linguistic embedding.

**Setting S2.** Only (RoBERTa) contextual embedding.

**Setting S3.** All linguistic features and (RoBERTa) contextual embedding.

**Setting S4.** Optimal linguistic features and (RoBERTa) contextual embedding.

**Setting S5.** GPT4.o zero shot learning.

**Setting S6.** Only (OpenAI) contextual embedding.

**Setting S1.** Only linguistic features.

|  | LR | XGB | RF | MLP | BiLSTM |
| --- | --- | --- | --- | --- | --- |
| Openness | 0.58 | 0.63 | 0.64 | 0.63 | 0.65 |
| Conscientiousness | 0.58 | 0.62 | 0.63 | 0.62 | 0.63 |
| Extraversion | 0.59 | 0.63 | 0.64 | 0.63 | 0.65 |
| Agreeableness | 0.62 | 0.65 | 0.66 | 0.66 | 0.66 |
| Neuroticism | 0.58 | 0.63 | 0.64 | 0.63 | 0.65 |

**Setting S2**. Only (RoBerta) contextual embedding.

|  | LR | XGB | RF | MLP | BiLSTM |
| --- | --- | --- | --- | --- | --- |
| Openness | 0.68 | 0.69 | 0.70 | 0.78 | **0.80** |
| Conscientiousness | 0.68 | 0.70 | 0.71 | 0.78 | **0.80** |
| Extraversion | 0.68 | 0.70 | 0.70 | 0.78 | **0.81** |
| Agreeableness | 0.70 | 0.71 | 0.72 | 0.79 | **0.82** |
| Neuroticism | 0.66 | 0.69 | 0.70 | 0.78 | **0.80** |

**Setting S3.** All linguistic features + contextual embedding.

|  | LR | XGB | RF | MLP | BiLSTM |
| --- | --- | --- | --- | --- | --- |
| Openness | 0.62 | 0.69 | 0.69 | 0.69 | 0.64 |
| Conscientiousness | 0.63 | 0.70 | 0.69 | 0.69 | 0.62 |
| Extraversion | 0.64 | 0.69 | 0.69 | 0.69 | 0.65 |
| Agreeableness | 0.66 | 0.71 | 0.70 | 0.72 | 0.66 |
| Neuroticism | 0.63 | 0.69 | 0.69 | 0.69 | 0.64 |

**Setting S4.** Optimal linguistic features + contextual embedding.

|  | LR | XGB | RF | MLP | BiLSTM |
| --- | --- | --- | --- | --- | --- |
| Openness | 0.66 | 0.69 | 0.69 | 0.77 | 0.81 |
| Conscientiousness | 0.67 | 0.70 | 0.69 | 0.77 | 0.81 |
| Extraversion | 0.67 | 0.70 | 0.69 | 0.77 | 0.81 |
| Agreeableness | 0.69 | 0.71 | 0.71 | 0.79 | 0.82 |
| Neuroticism | 0.66 | 0.69 | 0.69 | 0.77 | 0.81 |

**Setting S5.** GPT4.o zero shot learning.

**Note:** Zero shot learning doesn’t require additional training using another model. Hence, no result is presented for this setting.

**Setting S6.** Only (OpenAI) contextual embedding.

|  | LR | XGB | RF | MLP | BiLSTM |
| --- | --- | --- | --- | --- | --- |
| Openness | 0.68 | 0.69 | 0.70 | 0.78 | **0.82** |
| Conscientiousness | 0.68 | 0.70 | 0.71 | 0.78 | **0.82** |
| Extraversion | 0.68 | 0.70 | 0.70 | 0.78 | **0.83** |
| Agreeableness | 0.70 | 0.71 | 0.72 | 0.79 | **0.83** |
| Neuroticism | 0.66 | 0.69 | 0.70 | 0.78 | **0.82** |

**Abbreviations:**

LR: Logistic Regression

XGB: Extreme Gradient Boosting

RF: Random Forest

MLP: Multi Level Perceptron

BiLSTM: Bidirectional Long Short-Term Memory
